# Joint Probabilistic Data Association Filter with Unknown Detection Probability and Clutter Rate

**DOI:** 10.3390/s18010269

**Published:** 2018-01-18

**Authors:** Shaoming He, Hyo-Sang Shin, Antonios Tsourdos

**Affiliations:** School of Aerospace, Transport and Manufacturing, Cranfield University, MK43 0AL Cranfield, UK; Shaoming.He@cranfield.ac.uk (S.H.); a.tsourdos@cranfield.ac.uk (A.T.)

**Keywords:** multiple target tracking, joint probabilistic data association, multi-Bernoulli filter, unknown detection probability, unknown clutter rate

## Abstract

This paper proposes a novel joint probabilistic data association (JPDA) filter for joint target tracking and track maintenance under unknown detection probability and clutter rate. The proposed algorithm consists of two main parts: (1) the standard JPDA filter with a Poisson point process birth model for multi-object state estimation; and (2) a multi-Bernoulli filter for detection probability and clutter rate estimation. The performance of the proposed JPDA filter is evaluated through empirical tests. The results of the empirical tests show that the proposed JPDA filter has comparable performance with ideal JPDA that is assumed to have perfect knowledge of detection probability and clutter rate. Therefore, the algorithm developed is practical and could be implemented in a wide range of applications.

## 1. Introduction

There has been increasing attention on utilisation of small autonomous systems in military and civil applications. The issue is that the operations of these small autonomous systems are constrained by limited payload, as well as limited operation time and endurance. This has led to proliferation of lightweight, low-cost and energy efficient on-board sensors.

Reliable and autonomous target tracking is a fundamental aspect of situation awareness for autonomous systems [1,2,3]. Applying low-cost and lightweight on-board sensors in target tracking imposes additional challenges to the tracking problem since they are likely to contain some degree of uncertainties. Low-cost sensors are generally subject to a high clutter rate and low detection probability. When combined with the inherent uncertainties and complexity of the problem, the poor performance issue with these sensors could be significantly exacerbated in target tracking, especially in multi-target tracking (MTT) [1].

It is known that data association in MTT is a challenging issue. With a high clutter rate, data association in MTT becomes even more challenging. One of the most popular data association approaches is the multiple hypothesis tracking (MHT) proposed in [4,5]. Given high computational power, MHT is known as a powerful MTT algorithm to address the problem of measurement uncertainty. Data, in MHT, are processed recursively with a delayed logic scheme and MHT intentionally expands parent track hypotheses with received measurements to form child track hypotheses in decision-making trees. These child track hypotheses are subsequently assessed and the ones with low scores are pruned by thresholding to keep feasibility. Although MHT is known as a theoretically optimal Bayesian estimator, the exact solution of MHT is computationally intractable and thereby requires approximated implementations [6,7].

JPDA is another widely used one-scan data association method. The basic assumption of JPDA is that each measurement can originate from several candidate targets in the valid gate. Therefore, JPDA never make any hard decisions on the measurement-to-target association and is a soft decision filter. The key part of JPDA is to enumerate all feasible joint events to calculate the marginal probability for the track update. Compared with the multi-scan MHT, JPDA can achieve reasonable results at lower computational burden [8,9,10,11,12,13]. Since JPDA forms tracks out of the marginal measurement-to-track association distributions, it is known as a track-oriented approach.

Despite its advantages, the JPDA filter, similar to most multi-target tracking algorithms, requires the knowledge of detection probability and clutter rate in implementation. These two parameters are of great importance for the JPDA filter as they determine the final estimation performance. Significant mismatches of these two key parameters could result in erroneous tracking outputs. Although the detection probability, dependent on the sensors and the scenarios, can be tested via offline experiments, it might not be cost-effective and they are typically unavailable for low-cost sensors. Furthermore, under dynamic environmental conditions or detection approaches, the clutter rate generated by the same sensor might change in a great deal: for example, in image-based target tracking, measurements are extracted from various detection algorithms [14], ranging from simple background subtraction [15] to complicated deep learning approach [16]. Obviously, the clutter rate and detection probability are different for different detection methods. Therefore, the usual assumption on the full knowledge of these two key parameters for MTT in advance might not be realistic and how to accommodate the unknown detection probability and clutter rate is of critical importance in practice. Although this issue is tackled in previous random finite set (RFS) filters [17,18], they cannot preserve the labels of the targets and thus might have limited applications.

Motivated by the above observations, this paper aims to propose an enhanced version of JPDA that can accommodate the unknown detection probability and clutter rate. The proposed algorithm is derived by integrating a JPDA filter with Poisson point process (PPP) birth model and a multi-Bernoulli filter. Note that the study in this paper is based on our previous work [19]: this paper refines the algorithm developed in [19] and extends the performance evaluation through extensive empirical tests. Unlike previous works, where the clutter rate estimation is decoupled from the multi-object tracking [14,20,21,22], the proposed approach utilises a closed feedback loop structure based on the property of the posterior of the JPDA estimations. More specifically, the multi-target JPDA filter leverages the information of estimated detection probability and clutter rate provided by the multi-Bernoulli filter. In the meantime, the multi-Bernoulli filter uses the estimation outputs of the main JPDA tracker since the posterior of JPDA estimations can be modelled by a multi-Bernoulli RFS.

The performance of the proposed approach is evaluated through extensive numerical simulations. The simulation results demonstrate that the proposed algorithm has comparable performance with the ideal JPDA, which is assumed to have perfect knowledge of detection probability and clutter rate.

The rest of the paper is organised as follows. Section 2 presents the system models utilised. Then, Section 3 briefly introduces the JPDA filter and provide the analysis on the effect of detection probability as well as the clutter rate on the tracking performance. Next, the details of the proposed JPDA filter is given in Section 4. Finally, some experiments and conclusions are offered.

## 2. System Models and Assumptions

This section addresses the system models that are used in the following sections. Throughout the paper, we use the symbol *i* to refer to a target index and *j* to refer to a measurement index.

The set of target states and measurements received at scan *k* are, respectively, defined as

(1) $$X_{k}=\left\{x_{k}^{1},\ldots ,x_{k}^{N_{k}}\right\}\in X,\;\;\;Z_{k}=\left\{z_{k}^{0},z_{k}^{1},\ldots ,z_{k}^{M_{k}}\right\}\in Z$$

where $N_{k}$ denotes the number of targets at scan *k*, $x_{k}^{i}$ the *i*th target at scan *k*, $M_{k}$ the number of measurements received at scan *k*, $z_{k}^{j}\left(j\neq 0\right)$ the *j*th measurement received at scan *k*, $z_{k}^{0}$ the dummy measurement for convenient representation of miss detection. We assume that the temporal evolution of each target is independent of others and follows a Markov transition model $f\left(x_{k}\left|x_{k-1}\right.\right)$, then, the prediction of target state of scan *k* is governed by

(2) $$p_{k\left|k-1\right.}\left(x_{k}^{i}\right)=\int f\left(x_{k}^{i}\left|x_{k-1}^{i}\right.\right)p_{k-1\left|k-1\right.}\left(x_{k-1}^{i}\right)dx_{k-1}^{i}$$

As required for JPDA, we accept the assumption that each target can generate at most one measurement and each measurement can originate from at most one target. Each target-generated measurement is independent of each other and is detected with probability $P_{D}$ with measurement likelihood $p(z\left|x\right.)$.

The additional assumptions made in the paper are as follows:

- The clutter distribution is assumed to be unknown *a priori*. When there is no prior knowledge regarding the clutter measurements, the MTT problem generally assumes that the number of clutters or false alarms is locally Poisson distributed [23]. Therefore, this paper utilises a Poisson distribution as the clutter distribution. Clutters or false alarms are modelled by a local PPP with intensity $\lambda_{FA}\left(z\right)=N_{FA}/V_{s}$ with $N_{FA}$ being the average number of clutters of one scan and $V_{s}$ being the sensor volume. Clearly, if domain or environmental knowledge could be used to develop a more realistic clutter distribution in specific tracking tasks, the estimation performance could be improved.
- The detection probability is assumed to be independent of the target state in filter design. The detailed discussion will be presented in Section 4.1.

In standard JPDA filtering approach, a track is defined as a sequence of measurements that originate from the same target. The original JPDA filter assumes the number of targets is known a priori at each scan and utilise a wrapper heuristic M/N logic for track initiation and deletion. In this paper, we resort to a PPP model, similar to [24], for target birth instead of the heuristic M/N logic. In other words, we assume that target birth is modelled as a PPP with intensity $\lambda_{B}\left(x\right)$ and track is confirmed based on a random binary variable, target existence status, $e_{k}^{i}\in \left\{0,1\right\}$ with $e_{k}^{i}=1$ being the existence of the *i*th target and $e_{k}^{i}=0$ being non-existence. Then, measurements that either from new targets or false alarms can be modelled by a PPP with intensity $\lambda_{E}=\lambda_{B}\left(x\right)p\left(z\left|x\right.\right)P_{D}+\lambda_{FA}\left(z\right)$. Note that, by incorporating the PPP model into JPDA filter, the posterior multi-object PDF can then be represented by the multi-Bernoulli distribution. This fact enables the application of recently proposed multi-Bernoulli RFS to JPDA for joint detection probability and clutter rate estimation. Let $r_{k}^{i}=p\left(e_{k}^{i}=1\right)$ denote the existence probability of the *i*th target at scan *k*. Then, the time evolution of $r_{k}^{i}$ can be formulated by the Markov Chain One model [9] and the Markov transition probability matrix is determined by the survival probability $P_{S}$ [9].

## 3. Joint Probabilistic Data Association Filter and Analysis

In this section, we first briefly review the basics of JPDA filter for the completeness and then give an intuitive analysis of the effect of the detection probability and clutter rate.

### 3.1. Joint Probabilistic Data Association Filter

JPDA algorithm aims to calculate the marginalized association probability based on all possible joint events for data association. A joint event is an allocation of all measurements to all tracks. In JPDA, a feasible joint event is defined as one possible mapping of the measurements to the tracks such that: (1) each measurement (except for the dummy one) is assigned to at most one target; and (2) each target is uniquely assigned to a measurement. Let $\Theta_{k}=\left\{\theta_{k}^{i}\right\}$, $i\in \left\{1,2,\ldots ,N_{k\left|k-1\right.}\right\}$, denote the joint association event. For each pre-existed target $i\in \left\{1,2,\ldots ,N_{k\left|k-1\right.}\right\}$, $\theta_{k}^{i}\in \left\{0,1,\ldots ,M_{k}\right\}$ denotes the association event, where $\theta_{k}^{i}=j$ means the *j*th measurement is originated from the *i*th target and $\theta_{k}^{i}=0$ represents the dummy association in which the *i*th target is miss detected. JPDA assumes that each single association event is independent and the posterior of each target is characterised by a mixture as [5]

(3) $$p\left(x_{k}^{i}\left|e_{k}^{i}=1,Z_{k}\right.\right)=\sum_{\theta_{k}^{i}}p\left(x_{k}^{i}\left|\theta_{k}^{i},e_{k}^{i}=1,Z_{k}\right.\right)p\left(\theta_{k}^{i}\left|e_{k}^{i}=1,Z_{k}\right.\right)$$

Generally, propagation of the mixture distribution is computationally intractable due to the explosion of mixture terms. JPDA approximates this mixture term by a single Gaussian distribution through first moment matching method. More specifically, the state correction $x_{k\left|k\right.}^{i}$ for each pre-existed target $i\in \left\{1,2,\ldots ,N_{k\left|k-1\right.}\right\}$ and its corresponding covariance $P_{k\left|k\right.}^{i}$ of JPDA is obtained as

(4) $$\begin{array}{c} x_{k\left|k\right.}^{i}=\sum_{j=0}^{M_{k}}\beta_{j}^{i}x_{k\left|k\right.}^{i,j}\\ P_{k\left|k\right.}^{i}=\sum_{j=0}^{M_{k}}\beta_{j}^{i}\left\{P_{k\left|k\right.}^{i,j}+\left(x_{k\left|k\right.}^{i,j}-x_{k\left|k\right.}^{i}\right){\left(x_{k\left|k\right.}^{i,j}-x_{k\left|k\right.}^{i}\right)}^{T}\right\} \end{array}$$

where $x_{k\left|k\right.}^{i,j}$ denotes the target estimates from associating the *j*th measurement to target *i*, $P_{k\left|k\right.}^{i,j}$ the corresponding covariance. Note that $\left(x_{k\left|k\right.}^{i,j},P_{k\left|k\right.}^{i,j}\right)$ can be obtained from $p\left(x_{k}^{i}\left|\theta_{k}^{i},e_{k}^{i}=1,Z_{k}\right.\right)$. $\beta_{j}^{i}\overset{\Delta }{=}p\left(\theta_{k}^{i}=j\left|e_{k}^{i}=1,Z_{k}\right.\right)$ is the existence conditioned marginal association probability that the *j*th measurement is associated with the *i*th target.

The hypothesis-conditioned update $p\left(x_{k}^{i}\left|\theta_{k}^{i},e_{k}^{i}=1,Z_{k}\right.\right)$ can be calculated by standard Kalman filter algorithm. Therefore, the remaining part is how to obtain the marginal association probability $p\left(\theta_{k}^{i}\left|e_{k}^{i}=1,Z_{k}\right.\right)$ in a tractable way as this is the most computationally part.

The posterior distribution of the joint association event $p\left(\Theta_{k}\left|Z_{k}\right.\right)$ consists of two parts: miss detection $f_{m}$ and detection $f_{d}$ [9]. Therefore, one can imply that

(5) $$p\left(\Theta_{k}\left|Z_{k}\right.\right)\propto f_{m}\times f_{d}$$

where

(6) $$f_{m}\propto \prod_{i\in \left[N_{k\left|k-1\right.}\right],\theta_{k}^{i}=0}\left[1-r_{k\left|k-1\right.}^{i}+r_{k\left|k-1\right.}^{i}\left(1-P_{D}\right)\right]=\prod_{i\in \left[N_{k\left|k-1\right.}\right],\theta_{k}^{i}=0}\left[1-r_{k\left|k-1\right.}^{i}P_{D}\right]$$

(7) $$f_{d}\propto \prod_{i\in \left[N_{k\left|k-1\right.}\right],\theta_{k}^{i}=j}\left[\frac{p\left(z_{k}^{j}\left|x_{k}^{i}\right.\right)r_{k\left|k-1\right.}^{i}P_{D}}{\lambda_{FA}\left(z_{k}^{j}\right)+\lambda_{B}\left(z_{k}^{j}\right)p\left(z_{k}^{j}\left|x_{k}^{i}\right.\right)P_{D}}\right]$$

Note the term $1-r_{k\left|k-1\right.}^{i}$ in $f_{m}$ is for the non-existence of the *i*th target and $r_{k\left|k-1\right.}^{i}\left(1-P_{D}\right)$ is for the existence but non-detection of the *i*th target.

Although full enumeration is intractable in real applications due to the high demand of computational power, the marginal probability $p\left(\theta_{k}^{i}\left|Z_{k}\right.\right)$ can be approximated by m−best approximations, stochastic sampling or any other suitable approaches [10,11,12,25]. In this paper, we utilize the Gibbs sampling approach to approximate the marginal probability. This method enables fast calculation of the marginal probability with ignorable performance sacrifice. Detailed description of this algorithm can be found in our previous work [25]. For the completeness of the paper, a brief introduction to the Gibbs-JPDA implementation algorithm is provided in the Appendix.

After finding $p\left(\theta_{k}^{i}\left|Z_{k}\right.\right)$, the joint probability $p\left(\theta_{k}^{i},e_{k}^{i}=1\left|Z_{k}\right.\right)$ can be calculated using Bayesian rule as

(8) $$p\left(\theta_{k}^{i},e_{k}^{i}=1\left|Z_{k}\right.\right)=p\left(e_{k}^{i}=1\left|\theta_{k}^{i},Z_{k}\right.\right)p\left(\theta_{k}^{i}\left|Z_{k}\right.\right)$$

Based on Equations (5)–(7), the hypothesis-conditioned existence probability has the form as

(9) $$p\left(e_{k}^{i}=1\left|\theta_{k}^{i},Z_{k}\right.\right)\propto \left\{\begin{array}{cc} \frac{r_{k\left|k-1\right.}^{i}\left(1-P_{D}\right)}{1-r_{k\left|k-1\right.}^{i}+r_{k\left|k-1\right.}^{i}\left(1-P_{D}\right)},\; & \theta_{k}^{i}=0\\ 1,\; & \theta_{k}^{i}=j\\ \frac{\lambda_{{}_{B}}\left(x_{k}^{i}\right)p\left(z_{k}^{j}\left|x_{k}^{i}\right.\right)P_{D}}{\lambda_{{}_{FA}}\left(z_{k}^{j}\right)+\lambda_{{}_{B}}\left(x_{k}^{i}\right)p\left(z_{k}^{j}\left|x_{k}^{i}\right.\right)P_{D}},\; & \theta_{k}^{N_{k\left|k-1\right.}+j}=N_{k\left|k-1\right.}+j \end{array}\right.$$

Finally, the posterior probability of target existence $r_{k}^{i}$ and the existence-conditioned marginal probability $p\left(\theta_{k}^{i}\left|e_{k}^{i}=1,Z_{k}\right.\right)$ used in the estimation update can be calculated as

(10) $$r_{k\left|k\right.}^{i}=p\left(e_{k}^{i}=1\left|Z_{k}\right.\right)=\sum_{\theta_{k}^{i}}p\left(\theta_{k}^{i},e_{k}^{i}=1\left|Z_{k}\right.\right)$$

(11) $$p\left(\theta_{k}^{i}\left|e_{k}^{i}=1,Z_{k}\right.\right)=\frac{p\left(\theta_{k}^{i},e_{k}^{i}=1\left|Z_{k}\right.\right)}{p\left(e_{k}^{i}=1\left|Z_{k}\right.\right)}=\frac{p\left(e_{k}^{i}=1\left|\theta_{k}^{i},Z_{k}\right.\right)p\left(\theta_{k}^{i}\left|Z_{k}\right.\right)}{r_{k\left|k\right.}^{i}}$$

Using $r_{k\left|k\right.}^{i}$ and $p\left(\theta_{k}^{i}\left|e_{k}^{i}=1,Z_{k}\right.\right)$, we can now perform track update for the JPDA filter using Equations (3) and (4).

Note that, except for the updates of $N_{k\left|k-1\right.}$ pre-existed tracks, JPDA also creates $M_{k}$ new tracks for each measurement based on the birth model. After the track update, we use a similar approach, as shown in score-based approach [5], for track confirmation and deletion. A track is confirmed once its existence probability $r_{k}^{i}$ exceeds a pre-defined threshold and otherwise is still tentative and needs further test to be confirmed or deleted. Meanwhile, a track is immediately deleted if its existence probability $r_{k}^{i}$ is below a pre-defined threshold.

**Remark** **1.** *Note that the utilisation of PPP birth model for target existence estimation is similar to the idea of joint integrated probabilistic data association (JIPDA) [9,26]. However, the difference lies in that the JIPDA filter assumes a stationary or constant intensity of extraneous sources, which include unknown targets and false alarms while the proposed JPDA with PPP birth model dynamically estimate this intensity. More specifically, in JIPDA derivation, Equation (7) reduces to [9]*

(12) $$f_{d}\propto \prod_{i\in \left[N_{k\left|k-1\right.}\right],\theta_{k}^{i}=j}\frac{p\left(z_{k}^{j}\left|x_{k}^{i}\right.\right)r_{k\left|k-1\right.}^{i}P_{D}}{\lambda_{FA}\left(z_{k}^{j}\right)}$$

Therefore, the standard JIPDA ignores the influence of the unknown targets in marginalisation. Clearly, leveraging the PPP birth model is beneficial and more realistic in initialisation and calculating the marginal association probability.

### 3.2. Effect of the Detection Probability and Clutter Rate

This subsection analyses the effect of detection probability and clutter rate on the performance of JPDA filter provided that they are given in advance.

The effect of the detection probability $P_{D}$ on the estimation are two folds. On the one hand, it follows from Equations (6) and (7) that the the detection probability $P_{D}$ influences the relative ratio or weighting between the target miss detection $f_{m}$ and target detection $f_{d}$. Therefore, artificially lower detection probability will give more penalty on the target-absence $f_{m}$, leading to the delayed track maintenance. However, lower detection probability might be useful in holding targets through temporary fades. On the other hand, the fact that $\lambda_{E}=\lambda_{B}\left(x\right)p\left(z\left|x\right.\right)P_{D}+\lambda_{FA}\left(z\right)$ also reveals that the detection probability also affects the track initialization process. Lower detection probability will force longer tentative tracks before a confirmation or deletion decision is made based on the score logic. This, in turn, will increase the workload of the tracking filter as more tracks remain tentative, which is especially severe in a high-clutter scenario.

From Equation (7), one can note that the clutter rate $\lambda_{FA}$ also plays an important role in the JPDA algorithm. Higher clutter rate will give more penalty on the external source term, i.e., false alarms or new targets, $\lambda_{FA}\left(z_{k}^{j}\right)+\lambda_{B}\left(z_{k}^{j}\right)p\left(z_{k}^{j}\left|x_{k}^{i}\right.\right)P_{D}$ than the target-detection term $p\left(\chi_{k}^{i}\left|Z_{k}\right.\right)p\left(z_{k}^{j}\left|x_{k}^{i}\right.\right)P_{D}$. Therefore, artificially higher clutter rate provides an estimation result that more tracks will be considered as false alarms by the tracking system. Similarly, lower clutter rate usually generates overestimation of the real targets.

Consequently, significant mismatch of either detection probability $P_{D}$ or clutter rate $\lambda_{FA}$ will result in highly biased estimation. However, both $P_{D}$ and $\lambda_{FA}$ are difficult or not cost-effective to obtain in practice, especially for for low-cost sensors. Therefore, it is imperative to design an improved JPDA algorithm that can accommodate this issue.

## 4. Joint Probabilistic Data Association Filter with Unknown Detection Probability and Clutter Rate

In this section, we will propose a novel JPDA filter to adjust the unknown detection probability and clutter rate by leveraging the multi-Bernoulli filter.

### 4.1. Detection Probability and Clutter Rate Estimator

The recently proposed multi-Bernoulli filter [18,27,28,29] utilises the random finite set (RFS) theory to recursively propagate the posterior of multi-target state. This filter is known as a parametrised approximation of the Bayesian multi-target recursion. At each time instant *k*, the posterior PDF of the multi-target state is characterised as a multi-Bernoulli RFS. More specifically, each target is modelled as a single Bernoulli RFS, which is fully described by the existence probability $r_{k}^{i}$ and the probability density $p_{k}\left(x_{k}^{i}\right)$. A Bernoulli RFS is empty with probability $1-r_{k}^{i}$ and has only one element, whose distribution is described by $p(x_{i})$, with probability $r_{k}^{i}$. The multi-Bernoulli RFS is a simple union of $N_{k}$ single independent Bernoulli RFSs and thus can be completely characterised by the parameter set ${\left\{r_{k}^{i},p\left(x_{k}^{i}\right)\right\}}_{i=1}^{N_{k}}$. The recursive multi-Bernoulli filter is given by two steps as [27,28]:

(1) **Prediction step.** If the posterior multi-target density at time k−1 is characterised as a multi-Bernoulli RFS as $\pi_{k-1\left|k-1\right.}={\left\{r_{k-1\left|k-1\right.}^{i},p_{k-1\left|k-1\right.}\left(x_{k-1}^{i}\right)\right\}}_{i=1}^{N_{k-1\left|k-1\right.}}$, then, the prediction of the multi-target density is also a multi-Bernoulli as
  (13) $$\pi_{k\left|k-1\right.}={\left\{r_{B,k}^{i},p_{B,k}\left(x_{k}^{i}\right)\right\}}_{i=1}^{N_{B,k}}\cup {\left\{r_{k\left|k-1\right.,i},p_{k\left|k-1\right.}\left(x_{k-1}^{i}\right)\right\}}_{i=1}^{N_{k-1\left|k-1\right.}}$$
  where the first part denotes the multi-Bernoulli RFS of the birth model and the second part represents the prediction of existing targets with
  (14) $$r_{k\left|k-1\right.}^{i}=r_{k-1\left|k-1\right.}^{i}\langle p_{k-1\left|k-1\right.}\left(x_{k-1}^{i}\right),P_{S}\rangle$$
  (15) $$p_{k\left|k-1\right.}\left(x_{k-1}^{i}\right)=\frac{\langle f\left(x_{k}^{i}\left|x_{k-1}^{i}\right.\right),p_{k-1\left|k-1\right.}\left(x_{k-1}^{i}\right)P_{S}\rangle }{\langle p_{k-1\left|k-1\right.}\left(x_{k-1}^{i}\right),P_{S}\rangle }$$
  where $\langle \alpha ,\beta \rangle \overset{\Delta }{=}\int \alpha \left(x\right)\beta \left(x\right)dx$.
(2) **Update step.** If the predicted multi-target density at time *k* is characterised as a multi-Bernoulli RFS as $\pi_{k\left|k-1\right.}={\left\{r_{k\left|k-1\right.}^{i},p_{k\left|k-1\right.}\left(x_{k}^{i}\right)\right\}}_{i=1}^{N_{k\left|k-1\right.}}$, then, the update of the multi-target density is also a multi-Bernoulli as
  (16) $$\pi_{k}={\left\{r_{L,k}^{i},p_{L,k}\left(x_{k}^{i}\right)\right\}}_{i=1}^{N_{k\left|k-1\right.}}\cup {\left\{r_{U,k}\left(z\right),p_{U,k}\left(\cdot ,z\right)\right\}}_{z\in Z_{k}}$$
  where the legacy (miss detection) component ${\left\{r_{L,k}^{i},p_{L,k}\left(x_{k}^{i}\right)\right\}}_{i=1}^{N_{k\left|k-1\right.}}$ is given by
  (17) $$r_{L,k}^{i}=r_{k\left|k-1\right.}^{i}\frac{1-\langle p_{k\left|k-1\right.}\left(x_{k-1}^{i}\right),P_{D}\rangle }{1-r_{k\left|k-1\right.}^{i}\langle p_{k\left|k-1\right.}\left(x_{k-1}^{i}\right),P_{D}\rangle }$$
  (18) $$p_{L,k}\left(x_{k}^{i}\right)=p_{k\left|k-1\right.}\left(x_{k}^{i}\right)\frac{1-P_{D}}{1-\langle p_{k\left|k-1\right.}\left(x_{k}^{i}\right),P_{D}\rangle }$$
  and the measurement updated component is given by
  (19) $$r_{U,k}\left(z\right)=\frac{\sum_{i=1}^{N_{k\left|k-1\right.}}\frac{r_{k\left|k-1\right.}^{i}\left(1-r_{k\left|k-1\right.}^{i}\right)\langle p_{k\left|k-1\right.}\left(x_{k}^{i}\right),p\left(z\left|x_{k}^{i}\right.\right)P_{D}\rangle }{{\left(1-r_{k\left|k-1\right.}^{i}\langle p_{k\left|k-1\right.}\left(x_{k}^{i}\right),P_{D}\rangle \right)}^{2}}}{\lambda_{FA}\left(z\right)+\sum_{i=1}^{N_{k\left|k-1\right.}}\frac{r_{k\left|k-1\right.}^{i}\langle p_{k\left|k-1\right.}\left(x_{k}^{i}\right),p\left(z\left|x_{k}^{i}\right.\right)P_{D}\rangle }{1-r_{k\left|k-1\right.}^{i}\langle p_{k\left|k-1\right.}\left(x_{k}^{i}\right),P_{D}\rangle }}$$
  (20) $$p_{U,k}\left(x_{k}^{i},z\right)=\frac{\sum_{i=1}^{N_{k\left|k-1\right.}}\frac{r_{k\left|k-1\right.}^{i}P_{D}}{1-r_{k\left|k-1\right.}^{i}}p_{k\left|k-1\right.}\left(x_{k}^{i}\right)p\left(z\left|x_{k}^{i}\right.\right)}{\sum_{i=1}^{N_{k\left|k-1\right.}}\frac{r_{k\left|k-1\right.}^{i}}{1-r_{k\left|k-1\right.}^{i}}\langle p_{k\left|k-1\right.}\left(x_{k}^{i}\right),P_{D}\rangle }$$

The advantage of the multi-Bernoulli filter is that it utilises the mathematically sound RFS theory and thus directly avoids the data association process in multi-target tracking, leading to computational efficiency. However, the target identity or the target label cannot be maintained during the filtering recursion. This means that the multi-Bernoulli filter cannot be used to support the target label management. Therefore, if the label information is required for practice, the application of multi-Bernoulli filter is limited. Fortunately, as our aim is to estimate the clutter rate as well as the detection probability using the multi-Bernoulli filter, there is no need to manage the clutter identity.

To apply the multi-Bernoulli filter to estimate the clutter rate, we consider each false alarm as a target with its own transition model, birth model and death model [18]. This consideration is motivated by the fact that the target and the clutter have totally different dynamics and thus can be treated separately in estimation. The true targets and clutters in recursive filtering are therefore labelled by a discrete space $I=\left\{0,1\right\}$, where 1 is the label of real targets and 0 is for clutters. To further accommodate the detection probability estimation, we augment the state space as $\bar{X}=P\times X\times I$, where $P=\left[0,1\right]$ denotes the space of detection probability and × represents the Cartesian product. Since the new state space is a discrete one, the integration on this space is given by

(21) $$\int_{\bar{X}}f\left(\bar{x}\right)d\bar{x}=\int_{P\times X}f_{0}\left(P_{D},x\right)dP_{D}dx+\int_{P\times X}f_{1}\left(P_{D},x\right)dP_{D}dx$$

In reality, the detection probability is related to the target state. This fact makes the system state propagation/prediction of the augmented system intractable. To address this problem, we further assume that the detection probability is independent of the target state, i.e.,

(22) $$\begin{array}{c} f_{0}\left(P_{D},x\right)=f_{0}\left(P_{D}\right)f_{0}\left(x\right)\\ f_{1}\left(P_{D},x\right)=f_{1}\left(P_{D}\right)f_{1}\left(x\right) \end{array}$$

Note that the target detection probability used in JPDA is typically piecewise constant and thus the independence assumption is reasonable in filter design. Furthermore, the validation presented in the following section reveals that the proposed algorithm works well in the presence of time-varying detection probability.

In general, the clutter dynamics is highly dependent on the sensors. If we have some prior information regarding the sensors, we can utilise such information to build the clutter dynamics model. Otherwise, general models can be leveraged, e.g., Gaussian distribution and random walk. Similarly, if there is no prior information, a typical choice of the detection probability model is the well-known beta distribution $P_{D}∼\mathrm{Beta}\left(\alpha ,\beta \right)$ [30]. The beta distribution $\mathrm{Beta}\left(\alpha ,\beta \right)$ is a family of continuous distributions defined on the interval $\left[0,1\right]$ and has enough diversity to accommodate the changes of detection probability. By choosing different shaping parameters α and β, the beta distribution can accommodate different detection probability models.

By substituting the augmented state space into the original multi-Bernoulli filter, we can readily estimate the number of clutters at one scan ${\hat{N}}_{FA}$ by the summation of all confirmed targets with label 0 and the clutter rate estimation ${\hat{\lambda }}_{FA}$ is then given by ${\hat{\lambda }}_{FA}={\hat{N}}_{FA}/V_{s}$. As $P_{D}$ is part of the system states, it can directly obtained by the state estimation of multi-Bernoulli filter. Note that one can also apply the augmented system into JPDA to adapt to the unknown detection probability and clutter rate. However, the inherent data association process of JPDA will make practical implementation intractable.

### 4.2. Estimation Algorithm

**Figure 1:**
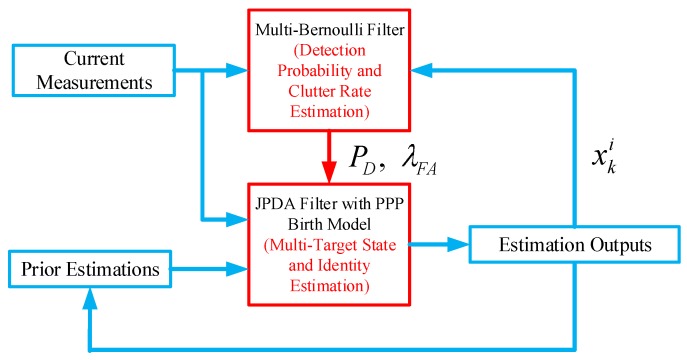
JPDA with unknown detection probability and clutter rate.

The proposed JPDA is shown in Figure 1. At each time instant *k*, we utilise the original JPDA with PPP birth model as the main tracker to provide the multi-target state estimation. The required detection probability and clutter rate is obtained from an one-step multi-Bernoulli filter. As discussed earlier, by incorporating the PPP model into JPDA, the posterior of JPDA estimation can be modelled as the multi-Bernoulli RFS and thus the estimated output of the JPDA is feedback to the multi-Bernoulli filter for its initialisation at every time instant. The advantage of the proposed parallel filtering scheme is that one can fully exploit the strengths of both approaches: using the JPDA for track management and obtaining fast estimation of detection probability as well as clutter rate by the multi-Bernoulli filter.

**Remark** **2.** *The computational complexity of the proposed estimation algorithm comes from two main parts: JPDA and multi-Bernoulli filter. Although the original marginalisation in JPDA is a #P-complete problem, leading to the fact that the computational time increases exponentially with respect to the number of targets, the approximation by Gibbs-sampling is a polynomial-time algorithm, as detailed in [25]. Additionally, since the RFS-based multi-Bernoulli filter directly avoids the typical data association procedure in multi-target tracking, it also runs in a polynomial time.*

## 5. Numerical Simulations

**Figure 2:**
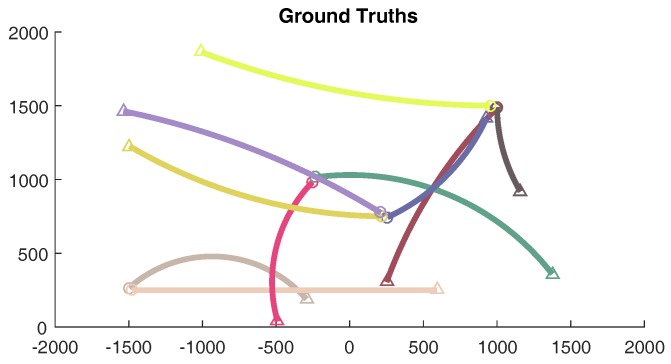
Ground truth of the considered scenario.

In this section, the effectiveness of the proposed JPDA filtering algorithm is investigated through numerical simulations in a cluttered environment. Our experiments explore a scenario, involving 10 manoeuvring targets with different birth time. The ground truth of the considered scenario is depicted in Figure 2.

### 5.1. Simulation Setup

The state vector contains planar position and velocity. We use the well-known constant velocity (CV) model for target prediction. The CV model is defined as

(23) $$x_{k}=F_{CV}x_{k-1}+Gw_{k-1}$$

with

(24) $$F_{CV}\overset{\Delta }{=}I_{2\times 2}⊗\left[\begin{array}{cc} 1 & T\\ 0 & 1 \end{array}\right],\;G\overset{\Delta }{=}\left[\begin{array}{cc} T^{2}/2 & 0\\ T & 0\\ 0 & T^{2}/2\\ 0 & T \end{array}\right]$$

where $I_{2\times 2}$ denotes the 2×2 identity matrix, *T* the sampling period, and $w_{k}∼N\left(\cdot ;0,\sigma_{v}^{2}\right)$ the Gaussian process noise.

It is assumed that we use a radar for multiple targets tracking. The target-generated nonlinear range and bearing measurements are modelled by

(25) $${\tilde{z}}_{k}=\left[\begin{array}{c} r_{k}\\ \alpha_{k} \end{array}\right]=\left[\begin{array}{c} \sqrt{{\left(x_{T,k}-x_{R}\right)}^{2}+{\left(y_{T,k}-y_{R}\right)}^{2}}\\ \arctan\left(\frac{y_{T,k}-y_{R}}{x_{T,k}-x_{R}}\right) \end{array}\right]+v_{k}$$

where $\left(x_{T,k},y_{T,k}\right)$ denotes target position, $\left(x_{R},y_{R}\right)$ radar position, and $v_{k}∼N\left(\cdot ;0,R_{k}\right)$ the Gaussian measurement noise with $R_{k}=\mathrm{diag}\left(\sigma_{r}^{2},\sigma_{a}^{2}\right)$.

**Figure 3:**
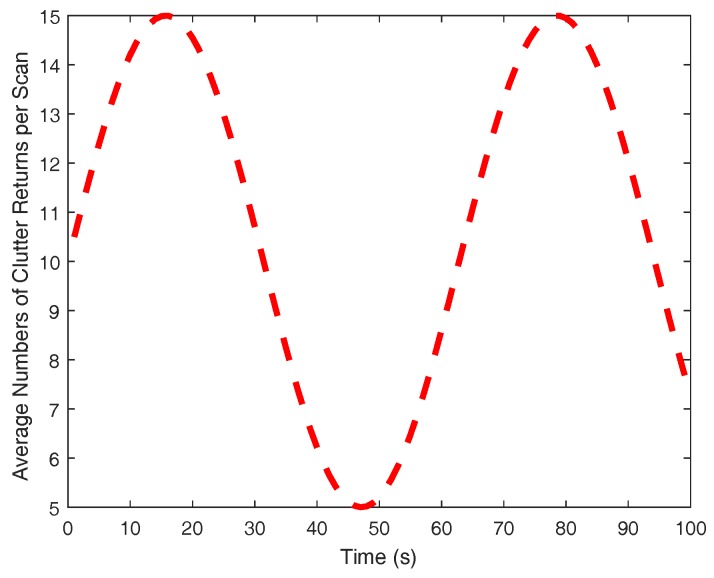
Clutter profile.

The clutter is assumed to be uniformly distributed in the surveillance region with its number being Poisson with $N_{FA}$ average returns at each scan. To validate the proposed algorithm in dynamic scenarios, $N_{FA}$ is given in a time-varying profile, as shown in Figure 3. Gating is performed with a threshold such that the gating probability is $P_{G}=0.999$. The detection probability $P_{D}$ peaks at the sensor origin with $P_{D}=0.95$ and exponentially decreases to 0.8 at the boundary of the sensing field-of-view. This setting is reasonable as the received signal strength decreases when the target is far away from the sensor. A tentative track is confirmed if the existence probability satisfies $r_{k}^{i}\geq 0.8$ and a confirmed track is deleted immediately once $r_{k}^{i}\leq 0.1$.

The optimal sub-pattern assignment (OSPA) distance metric [31] is considered here for overall performance evaluation, namely, cardinality and position estimation errors. Let *X* and *Y* be the position estimation set and true target position set, respectively. The cardinality of these two sets are *m* and *n*, respectively. Then, for c>0 and 1≤p<∞, the OSPA distance $d_{p}^{c}\left(X,Y\right)$ is defined as [31],

(26) $$d_{p}^{c}\left(X,Y\right)\overset{\Delta }{=}\left\{\begin{array}{cc} {\left[\frac{1}{n}\left(\min_{\pi \in \Pi_{n}}\sum_{i=1}^{m}d^{c}{\left(x_{i},y_{\pi \left(i\right)}\right)}^{p}+c^{p}\left(n-m\right)\right)\right]}^{1/p},\; & m\leq n\\ d_{p}^{c}\left(Y,X\right),\; & m>n \end{array}\right.$$

where $\Pi_{n}$ denotes the set of all permutations on $\left\{1,2,\ldots ,n\right\}$ for any positive integer *n*. $d^{c}\left(x_{i},y_{\pi \left(i\right)}\right)$ is the cut-off Euclidean distance between two vectors as $d^{c}\left(x_{i},y_{\pi \left(i\right)}\right)=\min\left(d\left(x_{i},y_{\pi \left(i\right)}\right),c\right)$ with $d\left(x_{i},y_{\pi \left(i\right)}\right)$ being the Euclidean distance. The order parameter *p* determines the sensitivity of $d_{p}^{c}\left(X,Y\right)$ in penalizing estimation outliers, while the cut-off parameter *c* determines the relative weighting of the penalties allocated to cardinality and localization errors. In all simulations, these two parameters are set as p=1, c=200.

### 5.2. Simulation Results

For the purpose of comparison, we also perform the ideal JPDA filter with perfect knowledge of detection probability and clutter rate, the following four non-ideal JPDA filters: (1) ideal $P_{D}$, $N_{FA}=1/10N_{FA}^{real}$; (2) ideal $P_{D}$, $N_{FA}=10N_{FA}^{real}$; (3) $P_{D}=0.6$, ideal $N_{FA}$; and (4) $P_{D}=0.3$, ideal $N_{FA}$.

**Figure 4:**
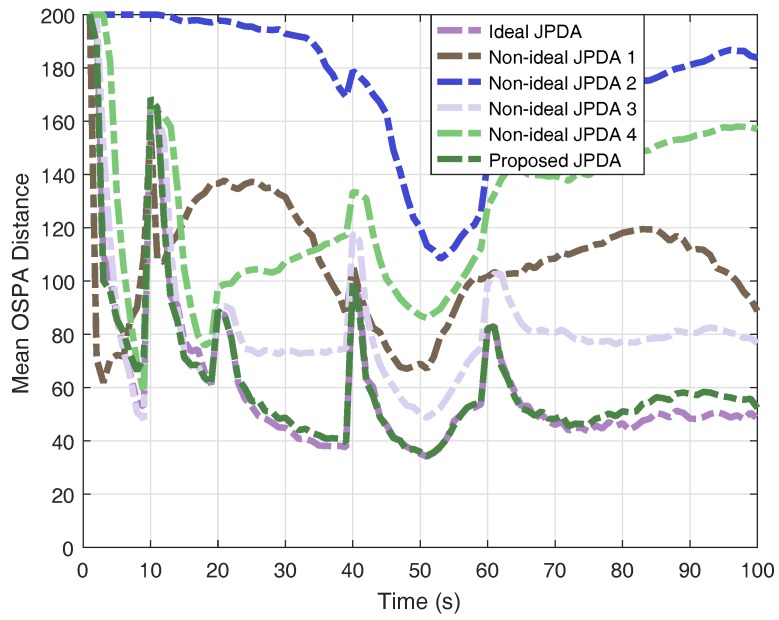
Monte-Carlo results of mean OSPA distance.

**Figure 5:**
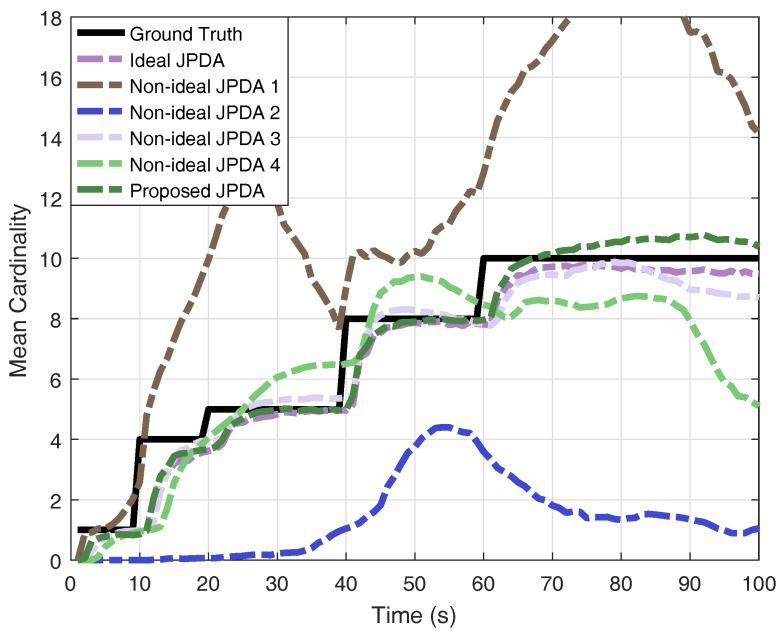
Monte-Carlo results of mean cardinality estimation.

The results of OSPA distance and cardinality estimation obtained by 200 Monte-Carlo runs are shown in Figure 4 and Figure 5. The obtained mean OSPA distances from different JPDAs in simulations are summarised in Table 1. The peaks of mean OSPA distance in Figure 4 are resulted from track confirmation for target birth. If the clutter rate is set lower than the real value, the JPDA filter overestimate the multi-target state and thus generating more ’confirmed’ targets, leading to larger OSPA distance (e.g., non-ideal JPDA filter 1). If the clutter rate is set higher than the real value, the JPDA filter gives an underestimation the multi-target state (e.g., non-ideal JPDA filter 2). Although the results, shown in Figure 4, reveal that the JPDA filter is somewhat robust against the detection probability (e.g., non-ideal JPDA filter 3), lower detection probability gives a delayed confirmation, as we stated earlier. Moreover, when the detection probability is highly mismatched with its real value (e.g., non-ideal JPDA filter 4), the estimation is unreliable. As a comparison, the results clearly verify that the proposed JPDA filter has comparable performance as the ideal JPDA filter by utilising the multi-Bernoulli estimator in a feedback loop.

## 6. Conclusions

This paper developed an enhanced version of JPDA by incorporating with the multi-Bernoulli filter to accommodate the unknown detection probability and clutter rate. By utilising the PPP birth in JPDA filter, the multi-target estimation output can be characterised as a multi-Bernoulli RFS. This enables the application of the multi-Bernoulli filter in a feedback loop to estimate the unknown detection probability and clutter rate. Simulation results confirm that the proposed algorithm exhibits the advantages of the original JPDA filter and can correct the biased estimations induced by the unknown detection probability and clutter. Under the condition where detection probability and clutter rate are difficult to be obtained, the proposed JPDA filter can be a practical solution, providing performance comparable to the ideal JPDA filter.

## Figures and Tables

**Table 1:** Mean OSPA distance of 200 Monte-Carlo runs.

	Ideal JPDA	JPDA 1	JPDA 2	JPDA 3	JPDA 4	Proposed JPDA
Mean OSPA distance	63.3483	107.3812	174.3893	81.1761	130.6579	64.2852

## Appendix A. Approximated Marginalisation by Gibbs Sampling

According to Equation (5), the posterior distribution of the joint event can be formulated as

(A1) $$p\left(\Theta_{k}\left|Z_{k}\right.\right)\propto \left(\prod_{i=1}^{N_{k\left|k-1\right.}}\varphi \left(\theta_{k}^{i}\right)\right)\left(\prod_{\left(i,i^{\prime }\right)\in E}\varphi_{c}\left(\theta_{k}^{i},\theta_{k}^{i^{\prime }}\right)\right)$$

where $\varphi \left(\theta_{k}^{i}\right)$ denotes the un-normalised PDA probability of $\theta_{i}$ given by

(A2) $$\varphi_{i}\left(\theta_{i}=j\right)\propto \left\{\begin{array}{cc} 1-r_{k\left|k-1\right.}^{i}P_{D},\; & j=0\\ \frac{p\left(z_{k}^{j}\left|x_{k}^{i}\right.\right)r_{k\left|k-1\right.}^{i}P_{D}}{\lambda_{FA}\left(z_{k}^{j}\right)+\lambda_{B}\left(z_{k}^{j}\right)p\left(z_{k}^{j}\left|x_{k}^{i}\right.\right)P_{D}},\; & j\neq 0 \end{array}\right.$$

and

(A3) $$\varphi_{c}\left(\theta_{k}^{i},\theta_{k}^{i^{\prime }}\right)=\left\{\begin{array}{cc} 0,\; & \theta_{k}^{i}=\theta_{k}^{i^{\prime }}>0\\ 1,\; & \mathrm{otherwise} \end{array}\right.$$

(A4) $$E=\left\{\left(i,i^{\prime }\right)\left|\exists i\in \left[N_{k\left|k-1\right.}\right],i^{\prime }\in \left[N_{k\left|k-1\right.}\right]\right.,i\neq i^{\prime }\right\}$$

which ensure that one measurement (except for the dummy measurement) can only be allocated to one target.

It is clear that determining the marginal joint association probability requires full enumeration of all possible joint association events, which is a well-known #P-complete problem. To tackle the combinatorial nature of this problem, our previous work suggested a sampling based, especially Gibbs sampling-based, algorithm [25]. The key idea of utilising Gibbs sampling in JPDA is to consider each joint association event $\Theta^{k}$ as a random variable that satisfies a distribution $\pi \left(\Theta_{k}\right)$. To ensure that the joint events with higher probability are more easily to be sampled, the sampling proposal $\pi \left(\Theta_{k}\right)$ is constructed proportional to the joint posterior probability as

(A5) $$\pi \left(\Theta_{k}\right)\propto \left(\prod_{i=1}^{N_{k\left|k-1\right.}}\varphi \left(\theta_{k}^{i}\right)\right)\left(\prod_{\left(i,i^{\prime }\right)\in E}\varphi_{c}\left(\theta_{k}^{i},\theta_{k}^{i^{\prime }}\right)\right)$$

The issue is that direct sampling from Equation (A5) is very difficult as enumerating all possible joint events is impossible for real-time applications. Therefore, Gibbs sampling to applied for generating the samples. The transition kernel from one joint event $\Theta_{k}=\left(\theta_{k}^{1},\ldots ,\theta_{k}^{N_{k\left|k-1\right.}}\right)$ to another joint event ${\bar{\Theta }}_{k}=\left({\bar{\theta }}_{k}^{1},\ldots ,{\bar{\theta }}_{k}^{N_{k\left|k-1\right.}}\right)$ is

(A6) $$\pi \left(\bar{\Theta }\left|\Theta \right.\right)=\prod_{m=1}^{N_{k\left|k-1\right.}}\pi_{m}\left({\bar{\theta }}_{k}^{m}\left|{\bar{\theta }}_{k}^{1},\ldots ,{\bar{\theta }}_{k}^{m-1},\theta_{k}^{m+1},\ldots ,\theta_{k}^{N_{k\left|k-1\right.}}\right.\right)$$

where $\pi_{m}$ can be obtained as

(A7) $$\begin{array}{c} \pi_{m}\left({\bar{\theta }}_{k}^{m}\left|{\bar{\theta }}_{k}^{1},\ldots ,{\bar{\theta }}_{k}^{m-1},\theta_{k}^{m+1},\ldots ,\theta_{k}^{N_{k\left|k-1\right.}}\right.\right)\\ \;\;\;=\frac{\pi \left({\bar{\theta }}_{k}^{1},\ldots ,{\bar{\theta }}_{k}^{m},\theta_{k}^{m+1},\ldots ,\theta_{k}^{N_{k\left|k-1\right.}}\right)}{\pi \left({\bar{\theta }}_{k}^{1},\ldots ,{\bar{\theta }}_{k}^{m-1},\theta_{k}^{m+1},\ldots ,\theta_{k}^{N_{k\left|k-1\right.}}\right)}\\ \;\;\;\propto \pi \left({\bar{\theta }}_{k}^{1},\ldots ,{\bar{\theta }}_{k}^{m},\theta_{k}^{m+1},\ldots ,\theta_{k}^{N_{k\left|k-1\right.}}\right)\\ \;\;\;\propto \varphi \left({\bar{\theta }}_{k}^{m}\right) \end{array}$$

“Proportion to” in Equation (A7) highlights the dependence of individual conditional distribution on ${\bar{\theta }}_{k}^{m}$, while rest parts are formed as the normalisation constant. Given the joint event $\Theta_{k}$, a joint event ${\bar{\Theta }}_{k}$ can be obtained by recursive sampling according to the following individual conditional distributions

(A8) $$\begin{array}{c} {\bar{\theta }}_{k}^{1}∼\pi_{1}\left({\bar{\theta }}_{k}^{1}\left|\theta_{k}^{2},\ldots ,\theta_{k}^{N_{k\left|k-1\right.}}\right.\right)\\ \;\;\;\vdots \\ {\bar{\theta }}_{k}^{m}∼\pi_{m}\left({\bar{\theta }}_{k}^{m}\left|{\bar{\theta }}_{k}^{1},\ldots ,{\bar{\theta }}_{k}^{m-1},\theta_{k}^{m+1},\ldots ,\theta_{k}^{N_{k\left|k-1\right.}}\right.\right)\\ \;\;\;\vdots \\ {\bar{\theta }}_{k}^{N_{k\left|k-1\right.}}∼\pi_{N_{k}}\left({\bar{\theta }}_{k}^{N_{k\left|k-1\right.}}\left|{\bar{\theta }}_{k}^{1},\ldots ,{\bar{\theta }}_{k}^{N_{k\left|k-1\right.}-1}\right.\right) \end{array}$$

Using Gibbs sampling, we could generate a sufficient number of samples of $\Theta_{k}$. From the samples, it is straightforward to approximate the marginal probability by the event occurrence.
